# A Tempest in A Ladle: The Debate about the Roles of General and Specific Abilities in Predicting Important Outcomes

**DOI:** 10.3390/jintelligence6020024

**Published:** 2018-04-19

**Authors:** Wendy Johnson

**Affiliations:** Department of Psychology, University of Edinburgh, 7 George Square, Edinburgh EH8 9JZ, UK; wendy.johnson@ed.ac.uk; Tel.: +44-0131-651-1304

**Keywords:** general cognitive ability, specific cognitive abilities, academic achievement, job performance, occupational attainment, health, longevity, situational specificity

## Abstract

The debate about the roles of general and specific abilities in predicting important outcomes is a tempest in a ladle because we cannot measure abilities without also measuring skills. Skills always develop through exposure, are specific rather than general, and are executed using different strategies by different people, thus tapping into varied specific abilities. Relative predictive validities of measurement formats depend on the purpose: the more general and long-term the purpose, the better the more general measure. The more specific and immediate the purpose, the better the closely related specific measure.

Excitabat enim fluctus in simpulo ut dicitur Gratidius.

For Gratidius raised a tempest in a ladle.

—Cicero, First century BCE, *De Legisbus*

In 2009, the *Journal of Research in Personality* published a Special Issue assessing the past and future of the famous person-situation debate in personality psychology. The issue, incorporating 81 personality psychologists as authors, included the usual editorial introduction, 38 empirical studies and evaluative essays, and a concluding perspective by Walter Mischel, whose 1968 book [1] is often considered to have originated the debate. One of the essays stood out as having assessed the question’s status most clearly, at least for me. It was the single-pager by Robert Hogan [2], who offered four reasons why the debate is ‘much ado about nothing’. His bottom line was that no one knows how to measure situations and everyone agrees that what a situation even ‘is’ depends on the perceptions of the people in them. However, these perceptions are always functions of those people’s personalities, so any situation definition would be affected by the very factors ‘theory’ says they influence.

I perceive the ‘situation’ of debate about the roles of general and specific abilities in predicting important outcomes to be a similar waste of time and resources. Of course this may be just an expression of my cranky personality—you can be the judge of that after I outline my reasons. Like Hogan’s, they do not constitute any kind of formal review, nor are they based on having run all the statistical tests that could be run. They are based, though, on reading a large share of the relevant research, doing many relevant statistical tests, and thinking hard about what we have done, what we can do with what we have, what might be missing, and what we could do to remedy that. At minimum, it is thus a dust-bowl empirically-based abstraction of an abstraction, and maybe a layer or two up from that.

There is massive confusion throughout the cognitive abilities research literature and assessment communities over which tasks measure skills and which measure abilities. I use ‘skill’ here to refer to performance clearly acquired through exposure and practice, and ‘ability’ to refer to some inherent capacity to acquire skills in general or particular kinds of skills, a distinction much easier to postulate than to articulate either conceptually or empirically with any clarity. This confusion is understandable because, much as we would like to, we have no assessments that measure purely either ability or skill, probably because there are no, and never could be, such tasks. All purported ability measures, especially those most often claimed otherwise such as the Raven, as evidenced by their large Flynn Effects, tap exposure and practice too, as can be seen by the substantial practice effects that show up just by administering the same test twice, as well as responses to task training. All purported skill measures such as typing or arithmetic tests also tap ability because even when exposure and opportunity to practice are closely controlled, individual differences in performance emerge. However, the primary reason there probably never could be such tasks is that babies can do almost nothing we recognize as cognitive—everything of that sort emerges through exposure and experience during ‘development’. It is not just a matter of someday identifying the relevant raw ‘biological’ material either: the brain is actively sculpted by experience, and genes all have environmentally mediated reaction norms.

Importantly, individual cognitive differences are often strategy-related, e.g., [3,4]. That is, people differ in the ways they do the same tasks, with some strategies being more effective than others. Part of any concept of general ability is the ability to figure out effective and efficient ways of approaching new tasks, so these differences do reflect this general ability. General ability is more than this, though: even when people are taught or told to use specific strategies and given the opportunity to practice before being tested, individual differences in performance remain [5]. This likely indicates differences in some kind of overall implementation capacity, but also indicates differences in which strategies ‘come easiest’ or ‘work best’, reflecting differences in what would be considered more specific abilities, as well as differences in prior exposure to relevant material.

The editors’ call for articles for this Special Issue of the *Journal of Intelligence* on what they term ‘the great debate’ about the relative merits of general and specific abilities in predicting real-world outcomes premised the debate on a consensus among researchers and practitioners that the ‘structure’ of human cognitive abilities can be modeled as a hierarchy consisting of a general ability factor that is associated with various levels of increasingly specific, more narrowly construed abilities. They noted appropriately, however, that this is about as far as any ‘consensus’ goes. Opinions differ as to just how the various ‘levels’ are related to each other, just what they might mean ‘biologically’, and how best to study them. In addition, every time anyone constructs a hierarchical model in a new battery, it comes out looking different from any one that the same person constructed in any other battery, not to mention different from the model some other researcher would construct in that same battery in that same sample. This is because the underlying factor-analytically-based methods are inherently subjective and because the relative associations among specific cognitive tasks vary both with sample specifics and with the specific other cognitive tasks in any battery.

Carroll [6] offered what I hope is the ultimate example of this variation. Try as he might across more than 460 datasets, he could not clearly carve out the natural ‘joints’ among specific abilities, nor even how many ‘levels’ of them there might be. My own work with the VPR model [7,8,9,10,11] shows this too: the specifics of the VPR model in each battery were different from every other one. This does not undermine the point of all that work, which was not to be specific about defining VPR model factors or specific abilities—the various verbal–perceptual and fluid-crystallized models all showed analogous differences. Rather, the point was to compare those two modeling perspectives and thus the relevance of their underlying structural premises. There, results were highly consistent, with the VPR model always fitting better. However, neither model ‘carved nature at its joints’ in any battery any better than Carroll had. This is because factor analysis spits back at us only what we put into it, and we have no tasks that uniquely measure any one particular ability or skill (see above).

At the same time, there is no question that we can design tasks that assess relatively specific, more narrowly construed abilities/skills. There is also no question that, if we have a good broad range of these, we can build yet another hierarchical model, extract its *g* factor in some nice broad sample, and this *g* factor will predict all kinds of important life outcomes from academic achievement to occupational attainment to longevity. Our model will also have a number of more specific factors, and none of them alone will ‘outshine’ the *g* factor in predicting life outcomes, as long as we keep those life outcomes rather broad. However, if we make the outcomes rather specific, and especially if we make them rather immediate, then those more specific abilities will predict the outcomes too, even after controlling the *g* factor, to the extent that the outcomes have content related to the assessed tasks and the outcomes are soon. Schmidt, Hunter, and Caplan [12] noted long ago that we had better also adjust for measurement error in all factors. Of course they were right, because we want to know about the predictive powers of whatever abilities/skills we are measuring, not those of the scores on whatever tasks we happened to dream up to assess them; so we do that too, and the more specific factors will still predict the outcomes, to the extent noted.

The bottom line for outcome predictors and selection practitioners is straightforward: if you want to predict a rather specific outcome happening rather soon, such as next year’s school grades in a specific subject, or seek an employee who can perform productively tomorrow or at least this week, assess specific content/job-related tasks. However, if you are going for long-term prediction and, on the job, are prepared to invest in training and offer incentives that will be needed to keep the employee around to make good on that investment, go for general cognitive ability. For your purposes, you can leave the question of to what degree you just assessed accrued cognitive skills and/or some kind of inherent capacity to the researchers. That one is rather thorny and inevitably developmental rather than merely structural, but the debate over the relative importance of general and specific abilities in predicting important life outcomes is a tempest in a ladle that has run its course.
